# Enhanced energy storage in relaxor (1-x)Bi_0.5_Na_0.5_TiO_3_-xBaZr_y_Ti_1-y_O_3_ thin films by morphotropic phase boundary engineering

**DOI:** 10.1038/s43246-024-00730-x

**Published:** 2025-01-14

**Authors:** Herbert Kobald, Alexander M. Kobald, Ivana Panzic, Marco Deluca

**Affiliations:** 1 Materials Center Leoben Forschung GmbH, Leoben, Austria; 2 Silicon Austria Labs GmbH, Graz, Austria

**Keywords:** Materials science, Energy science and technology

## Abstract

Perovskites at the crossover between ferroelectric and relaxor are often used to realize dielectric capacitors with high energy and power density and simultaneously good efficiency. Lead-free Bi_0.5_Na_0.5_TiO_3_ is gaining importance in showing an alternative to lead-based devices. Here we show that (*1-x*)Bi_0.5_Na_0.5_TiO_3_ – *x*BaZr_*y*_Ti_*1-y*_O_3_ (best: 0.94Bi_0.5_Na_0.5_TiO_3_ -0.06BaZr_0.4_Ti_0.6_O_3_) shows an increase of recoverable energy density and electric breakdown upon chemical substitution. In thin films derived from Chemical Solution Deposition, we observed that polarization peaks at the morphotropic phase boundary at *x* = 0.06. While Zr substitution results in reduced polarization, it enhances both efficiency and electric breakdown strength, ultimately doubling the recoverable energy density and the metallization interface by lowering surface roughness. Our dielectric capacitor shows <3% deviation of energy properties over 10^6^ cycles. A virtual device model of a multilayer thin film capacitor (7.25 mJ recoverable energy) was used to compare its performance to already in use multilayer ceramic capacitors.

## Abstract (web summary)

Lead-free perovskites at the crossover of ferroelectric and relaxor behavior are promising for use in dielectric capacitors. Here, (1-x)Bi_0.5_Na_0.5_TiO_3_-xBaZr_y_Ti_1-y_O_3_ thin films fabricated via chemical solution deposition are used in capacitors with good energy storage capability.

## Introduction

To efficiently power wireless and energy-autonomous smart sensor systems e.g., in the field of Internet of Things (IoT) and Industrial IoT applications, the energy storage device is a crucial element. In fact, it needs to possess at the same time high power density and high energy density, to withstand fast changes in energy intake and demand, and to supply energy during long harvesting-off times, respectively. Given these requirements, batteries and electrochemical supercapacitors often fall short due to their slower response times, making dielectric (ferroelectric) capacitors more appealing as energy storage elements for such applications. To enhance their energy storage capabilities, current research focuses on combining thin film technology with chemical modifications of ferroelectric materials to minimize hysteretic losses. Lead-free relaxor perovskites are increasingly important for energy storage capacitor devices, and they are starting to replace the commonly used lead-based ones in many state-of-the-art applications^1–4^. In these non-linear dielectric materials, the recoverable energy density *W*_*rec*_ is the area between the polarization vs. electric field hysteresis loop (PE-loop) and the polarization axis. It can be written as the following integral over the polarization *P*, whereas *P*_*r*_ is the remnant polarization, *P*_*s*_ is the saturation polarization (i.e., at maximum applied electric field) and *E* stands for the electric field^2,5,6^:

1 $${W}_{{\!\!rec}}={\int }_{{\!\!\!\!\!P}_{r}}^{{P}_{s}}{EdP}$$

**Fig. 1:**
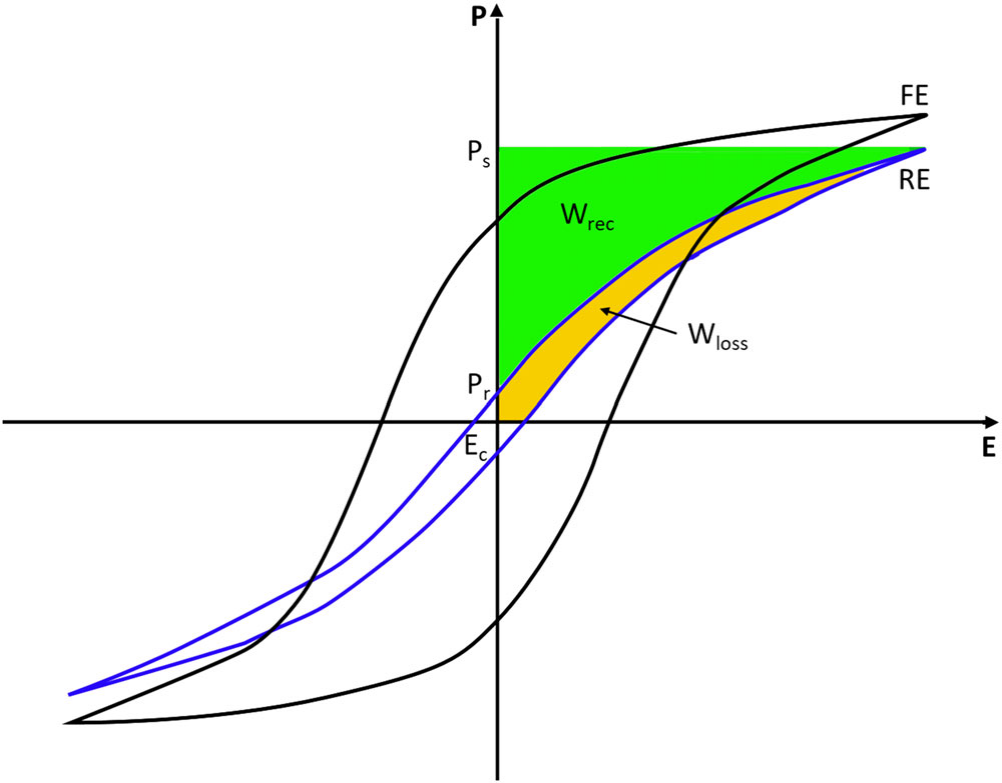
Scheme of polarization hysteresis loop. Schematic polarization vs. electric field hysteresis loop (PE-loop) of a classical ferroelectric (FE) and a relaxor material (RE) with indication of *W*_*rec*_ and *W*_*loss*_, remnant polarization (*P*_*r*_) and maximum/saturation polarization (*P*_*s*_).

Figure 1 schematically shows this relation and illustrates the PE-loops of a classical ferroelectric material (FE) and a relaxor ferroelectric (RE). Although FEs have a very high saturation polarization, their energy storage properties are not ideal, as they show high remnant polarization. This is associated with the strong energy dissipation in FEs, related to elastic energy dissipation by ferroelectric domain switching. Reaching a so-called relaxor state by chemical modification of ferroelectrics, the overall energy storage properties can be improved significantly. The reason for the enhanced energy storage density is the substituent-induced disruption of the long-range ferroelectric ordering in ferroelectrics by creating polar nanoregions in a non-polar matrix (or vice-versa) which decreases the remnant polarization, slims down the PE-loop and therefore greatly reduces the hysteretic losses induced by domain switching^5,7^.

The starting relaxor perovskite material Bi_0.5_Na_0.5_TiO_3_-BaTiO_3_ (BNT-BT) used in this work offers the aforementioned properties and is therefore a good candidate for energy storage capacitor applications^8,9^. BNT-BT has a morphotropic phase boundary (MPB) from rhombohedral to tetragonal phase at around 6 mol% of BaTiO_3_ content (i.e., BNT-6BT). Near this transition, the material demonstrates enhanced dielectric and energy storage properties. Therefore, exploring and fine-tuning the composition around the MPB is essential for optimizing its performance as an energy storage capacitor^10,11^. In addition, a key aspect to enhance the energy storage properties and their long-term stability is to reduce leakage currents by introducing up to 1 mol% of Mn at the B-site. Mn acts, in fact, as a dopant owing to its multiple valence states, by creating charge traps in the band gap that can attract extra electrons from oxygen vacancies^5,12^.

The further tailoring of the energy storage material by substituting Ti with Zr results in a strain-induced disruption of long-range ferroelectric ordering due the different ionic radii of Ti (0.605 Å) and Zr (0.72 Å)^13^. This slims down the polarization hysteresis loops, reduces the remnant polarization, diminishes the coercive field and thus enhances the overall energy storage properties of the (*1-x*)Bi_0.5_Na_0.5_TiO_3_-*x*BaZr_*y*_Ti_*1-y*_O_3_ (BNT-BZT) material^11,14^.

In this work, we utilize thin film technology, as thin films can be precisely engineered to have a lower defect density compared to bulk materials. This results in enhanced and more consistent electrical performance. The reduced thickness of the films also allows for the application of higher electric fields before breakdown and enlarges the capacitance per unit area as it is indirect proportional to the distance. When producing capacitor-like structures as done in this work, both aspects lead to an increase of energy storage properties. Another benefit of this technique is that it enables the miniaturization of capacitors which is especially needed in state-of-the-art electronic devices, such as IoT sensors^15^.

For the production of perovskite thin films many different deposition techniques can be used. Using Chemical Solution Deposition (CSD) offers some significant advantages. First of all, CSD is very cost-effective, especially when compared with other deposition methods such as physical vapor deposition or molecular beam epitaxy. Typically, the needed equipment is less costly, the precursor materials are less expensive, and the deposition rate is higher. CSD also offers the benefit of being highly flexible in production of materials with finely-tuned compositions, since it offers precise composition control by adjusting the precursor solutions’ chemistry. This degree of control enables rapid compositional screening and customization of material properties to meet precise specifications. Additional advantages include excellent industrial scalability and the potential for extensive automation^16,17^.

Here, we produced a capacitor-like structure with BNT-BT and BNT-BZT relaxor perovskite materials as dielectric layer on platinized Si substrates as these are highly compatible with the CMOS (complementary metal-oxide-semiconductor) technology used in IoT sensors. Au is used as top electrodes. In the end, a working capacitor structure with different compositions of BNT-BT and BNT-BZT as dielectric layers is built up and subjected to thorough structural and electrical characterization, including fatigue and long-term stability. The cross-over from FE to RE was found in CSD thin films at *x* = 0.06 with combined Raman spectroscopy and grazing incidence X-ray diffractometry (GI-XRD) analysis. The increase in breakdown strength could be assigned to the increased microstructure as shown by Atomic Force Microscopy (AFM) and Scanning Electron Microscopy (SEM). The composition showing the best properties with *W*_*rec*_ = 29 J cm^−3^ and an efficiency of >60% (0.94Bi_0.5_Na_0.5_TiO_3_ -0.06BaZr_0.4_Ti_0.6_O_3_; BNT-6BZT40) is selected and a virtual device model is built using its characteristics, in order to demonstrate that the performance of this composition as a multilayer thin film capacitor (MTFC) provides sufficient energy (7.25 mJ) to an energy autonomous IoT sensor node.

## Results and discussion

### Structural analysis

**Fig. 2:**
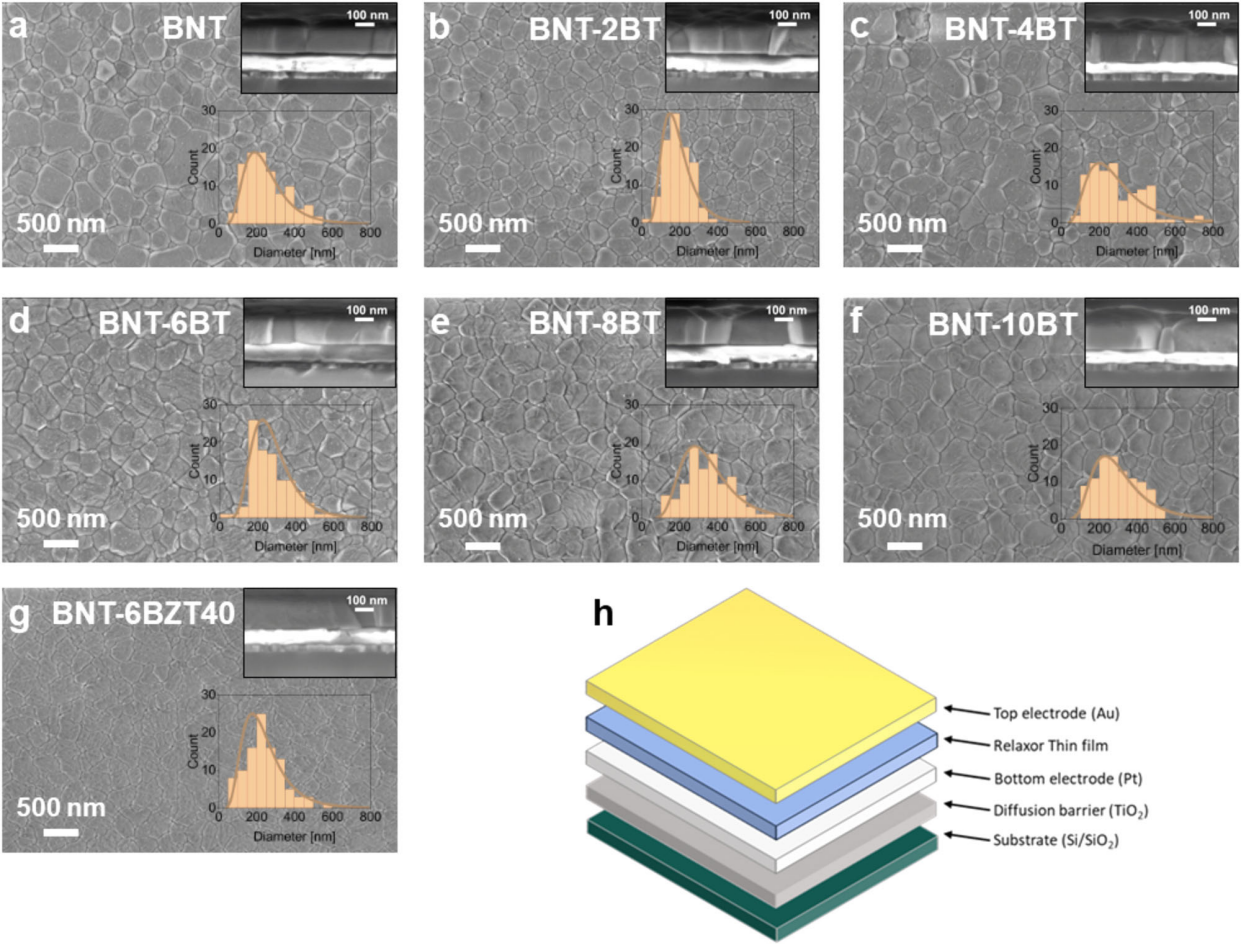
SEM top view and cross-section. **a** BNT, **b** BNT-2BT, **c** BNT-4BT, **d** BNT-6BT, **e** BNT-8BT, **f** BNT-10BT, and **g** BNT-6BZT40 thin films performed with Scanning Electron Microscopy and **h** schematic illustration of a metal-insulator-metal (MIM) capacitor structure as used in this work.

Figure 2a–g show the SEM analysis of the surfaces from the BNT-100*x*BT (with *x* = 0, 0.02, 0.04, 0.06, 0.08 and 0.10) and BNT-6BZT40, with the cross-section and the grain size distribution as inserts. From the surface-view it can be seen that all samples show high density with no visible porosity or cracks. The grain size distribution is uniform for all samples following a lognormal distribution. A slight tendency of increasing distribution width with increasing BaTiO_3_ substitution can be noticed. The average grain size - determined from the median of the lognormal distribution - shows little variation with substitution of BaTiO_3_ (cf. Fig. 2a–f) and Zr (cf. Fig. 2g). For other material systems, e.g., NaNbO_3_, it was found that small amounts of BaTiO_3_ substitution already led to a change in nucleation mechanism, leading towards a strongly decreased grain size and granular microstructure^6^. However, Bi_0.5_Na_0.5_TiO_3_ seems to withstand the substitution up to 10 mol% BaTiO_3_ without significant changes in both grain size and growth mode (columnar growth seen in the cross-sections). The reason might be that the start of nucleation from Bi_0.5_Na_0.5_TiO_3_ is at close temperatures compared to the intermediate phases of BaTiO_3_, therefore, no large increase in nucleation sites is expected. All films of both series have an overall film thickness of around 200 nm. A schematic of the metal-insulator-metal (MIM) capacitor architecture used to measure the electrical measurements of the thin films (cf. Section 2.2) is shown in Fig. 2h.

**Fig. 3:**
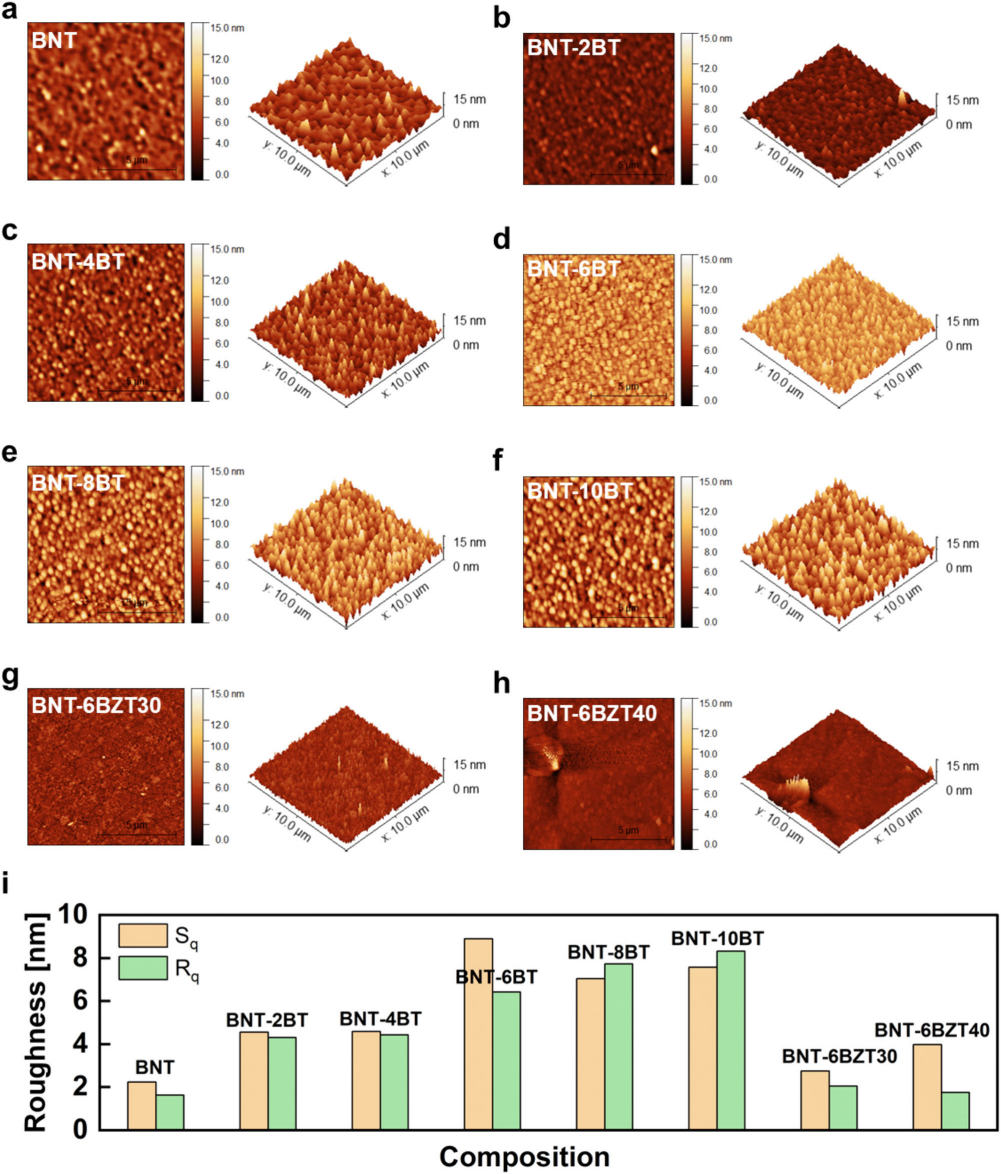
2D and 3D atomic surface morphology (AFM) images. **a**–**f** BNT-100*x*BT, **g**, **h** BNT-6BZT100*y* (*y* = 0.3, 0.4). **i** Analysis of root mean square line (*R*_*q*_) and area (*S*_*q*_) roughness.

The energy storage performance of a capacitor is related to the electric breakdown, which is strongly governed by the microstructure (cf. Equation(1)). Therefore, further microstructural analysis was performed by AFM in order to attain better spatial resolution than SEM in analyzing surface roughness and defects. In fact, the surface roughness of the dielectric layer significantly impacts the interface with the metallization layers and must be minimized to ensure reliable processing and high-quality stacking of multiple layers. In Fig. 3 the surface topography measured by AFM is shown. Smooth surfaces with a low roughness below 10 nm for all samples were found. The root mean square line (*R*_*q*_) and area (*S*_*q*_) roughness are plotted for all samples in Fig. 3i. *R*_*q*_ and *S*_*q*_ were found to increase with increasing BaTiO_3_ substitution in BNT-100*x*BT from 1.63 nm and 2.24 nm for pure BNT to 8.32 nm and 7.58 nm in BNT-10BT, respectively. However, Zr substitution significantly decreased *R*_*q*_ and *S*_*q*_ from 6.43 nm and 8.90 nm (BNT-6BT) to 1.75 nm and 3.98 nm (BNT-6BZT40), leading to a flatter and smoother surface. Therefore, Zr substitution can be effectively used to counteract the increased surface roughness from BaTiO_3_ substitution.

**Fig. 4:**
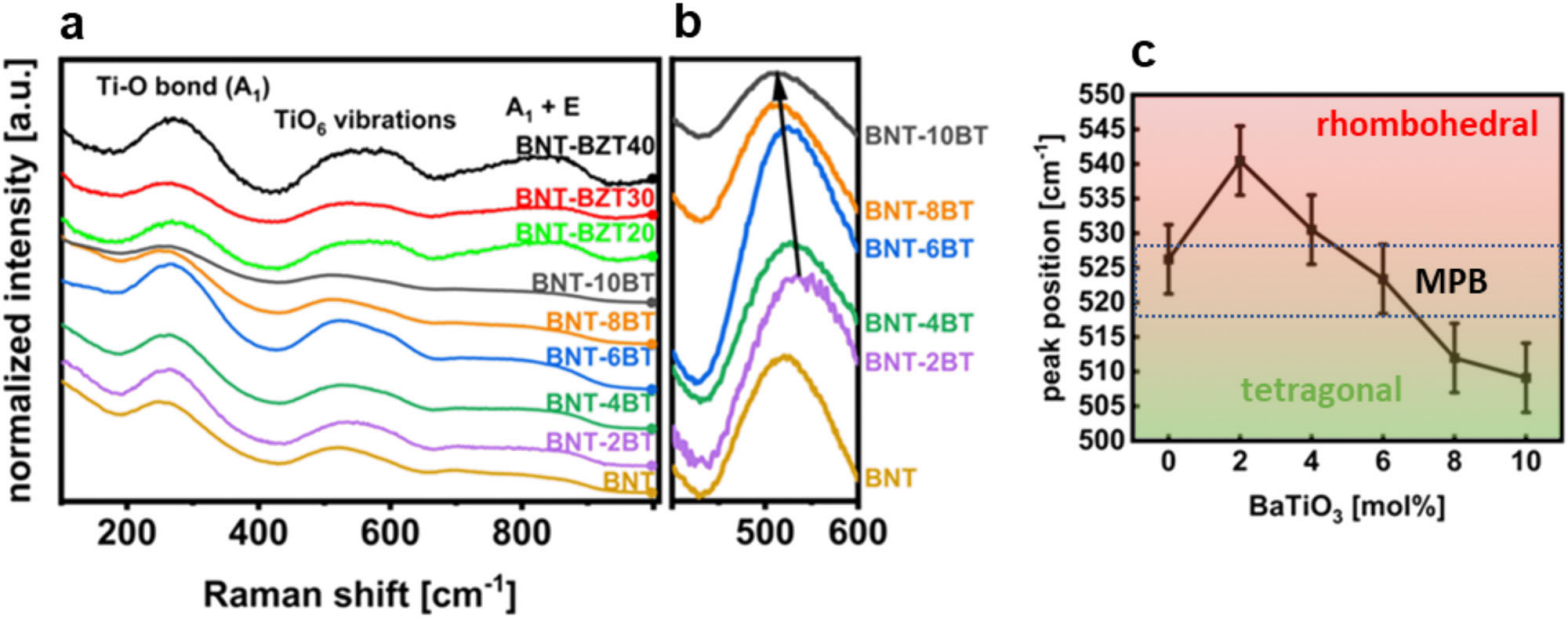
Raman spectra across varied BT and BZT substitution. **a** BNT-100*x*BT (with *x* = 0, 0.02, 0.04, 0.06, 0.08 and 0.10) and BNT-6BZT100*y* (with *y* = 0, 0.2, 0.3, 0.4) thin films at room temperature, **b** zoomed-in area of the peak-position around 540 cm^−1^ that marks the morphotropic phase transition from rhombohedral to tetragonal of the BNT-100*x*BT thin films, and **c** corresponding peak-positions of the rhombohedral and tetragonal peaks of the BNT-100*x*BT thin films with estimated error bars.

Raman spectroscopy was performed at room temperature. Figure 4 is a combined graph of the Raman spectra of the BNT-100*x*BT thin films as well as the BNT-6BZT100*y* thin film series. Within the spectra, the following main regions can be detected: a feature around 280 cm^−1^, associated to Ti-O vibrations; a feature around 450–700 cm^−1^ arising from TiO_6_ octahedra vibrations, and the high-frequency region above 700 cm^−1^ that is related to the longitudinal optical A1 and E overlapping bands^18,19^. All measured spectra show the typical shape of Bi_0.5_Na_0.5_TiO_3_-based compositions with very broad peaks, indicating high translational disorder. Figure 4b highlights the peak position around 540 cm^−1^ of the BNT-100*x*BT spectra, indicating a visible shift of this vibration with composition, which is assigned to the TiO_6_ octahedral vibration. Increasing BaTiO_3_ substitution, in fact, moves the 540 cm^−1^ peak towards lower wavenumbers. This is due to the transition from rhombohedral Bi_0.5_Na_0.5_TiO_3_ towards a tetragonal BaTiO_3_ phase^10^, leading to a distortion in the unit cell and changed lattice constants, which impact on the vibrational energies. Both rhombohedral and tetragonal phases of BNT-BT have characteristic peaks that are separated well enough to allow to differentiate between these phases at 541 cm^−1^ (rhombohedral) and 488 cm^−1^ (tetragonal)^19^. Furthermore, the tetragonal peak of BaTiO_3_ is located at 520 cm^−1^. Figure 4c displays the corresponding peak position of the convoluted peak resulting from these modes. A clear trend of shifting from the high-wavenumber position of the rhombohedral portion of the convoluted peak to the low-wavenumber position of the tetragonal portion is observed. This clearly indicates that the intensity of the rhombohedral mode decreases whereas that of the tetragonal mode increases. If both peaks of Bi_0.5_Na_0.5_TiO_3_ are weighted equally, a peak position of around 515 cm^−1^ should mark the phase transition area with coexistence of both phases. The overall measured convoluted peak, however, is shifted to higher wavenumbers because of the additional influence of the tetragonal BaTiO_3_ peak at 520 cm^−1^. 4 mol% and 8 mol% BNT-100*x*BT show already peak shifts of ±10 cm^−1^, which emphasizes the narrow range of the diffuse phase transition region. These findings show that also for thin films the MPB can be found at (6 ± 1) mol% BaTiO_3_ substitution^10,20^.

**Fig. 5:**
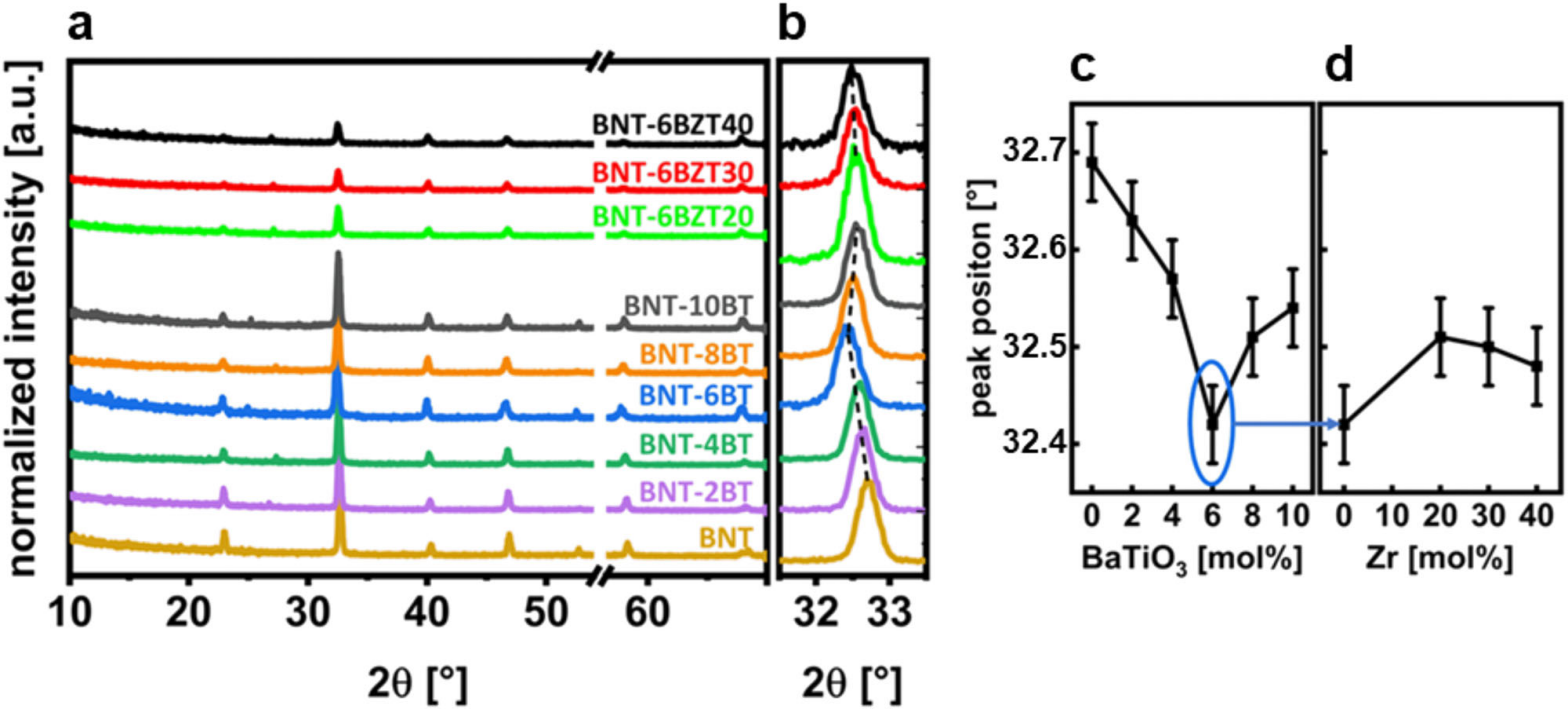
GI-XRD characterization. **a** Patterns of BNT-100*x*BT (with *x* = 0, 0.02, 0.04, 0.06, 0.08 and 0.10) and BNT-6BZT100*y* (with *y* = 0, 0.2, 0.3, 0.4) thin film series with removed substrate peak on x-axis (break tab), **b** zoomed-in position at around 32.5° (110), **c** variation of peak position around 32.5° (110) of BNT-100*x*BT series over BaTiO_3_-substitution and **d** variation of peak position around 32.5° (110) of BNT-6BZT100*y* series of Zr substitution with estimated error bars.

GI-XRD was performed to further investigate the phase transition area as well as to check phase purity. In Fig. 5 the patterns of the GI-XRD measurements are presented combined for BNT-100*x*BT and BNT-6BZT100*y*. The diffractograms show a pseudo-cubic perovskite structure with no major secondary phases visible^21^. The (110) peak of the thin films shows the highest intensity and is therefore zoomed-in to evaluate a shift in the peak position, as shown in Fig. 5b. For the BNT-100*x*BT series, an obvious shift with a kink at *x* = 0.06 is found, whereas the peak shifts for the BNT-6BZT100*y* unidirectional to lower *2θ* (Fig. 5c and d, respectively). Again, the morphotropic phase boundary is well visible at *x* = 0.06 with the lowest *2θ* and a very steep increase of *2θ* around it in both directions. Zr substitution, using BNT-6BT as starting composition, first increases the *2θ* value, whereas further Zr substitution leads to a subtle linear decrease of the (110) peak position. It is noticeable that the effect of BaTiO_3_ substitution affects the *2θ* value much stronger compared to Zr substitution. The reason is likely the more severe influence of the phase transition and boundary region on the lattice constants between tetragonal and rhombohedral than the change of effective ionic radii from Ti to Zr^22^. The GI-XRD results correlate well with the findings of Raman spectroscopy and literature^23^, both indicating the MPB at *x* = 0.06. Starting BNT was assigned to rhombohedral perovskite phase space group R3c (C6 3n) of Bi_0.5_Na_0.5_TiO_3_ (ICDD PDF#01-085-053), which is common for room temperature BNT. However, the rhombohedral and tetragonal structure of BNT are very close to cubic, resulting in symmetrical crystal shape. With the increase of BT in the system we see the shift to tetragonal space group P4mm (C1 4n) (ICDD PDF#70-4760). It is important to note the difficulty in accurately determining the phase ratio in the MPB region (in the compositional range of approximately *x* = 0.06) due to the broadening of XRD peaks, which is caused by phase coexistence and low crystallinity^21^.

### Electrical analysis

**Fig. 6:**
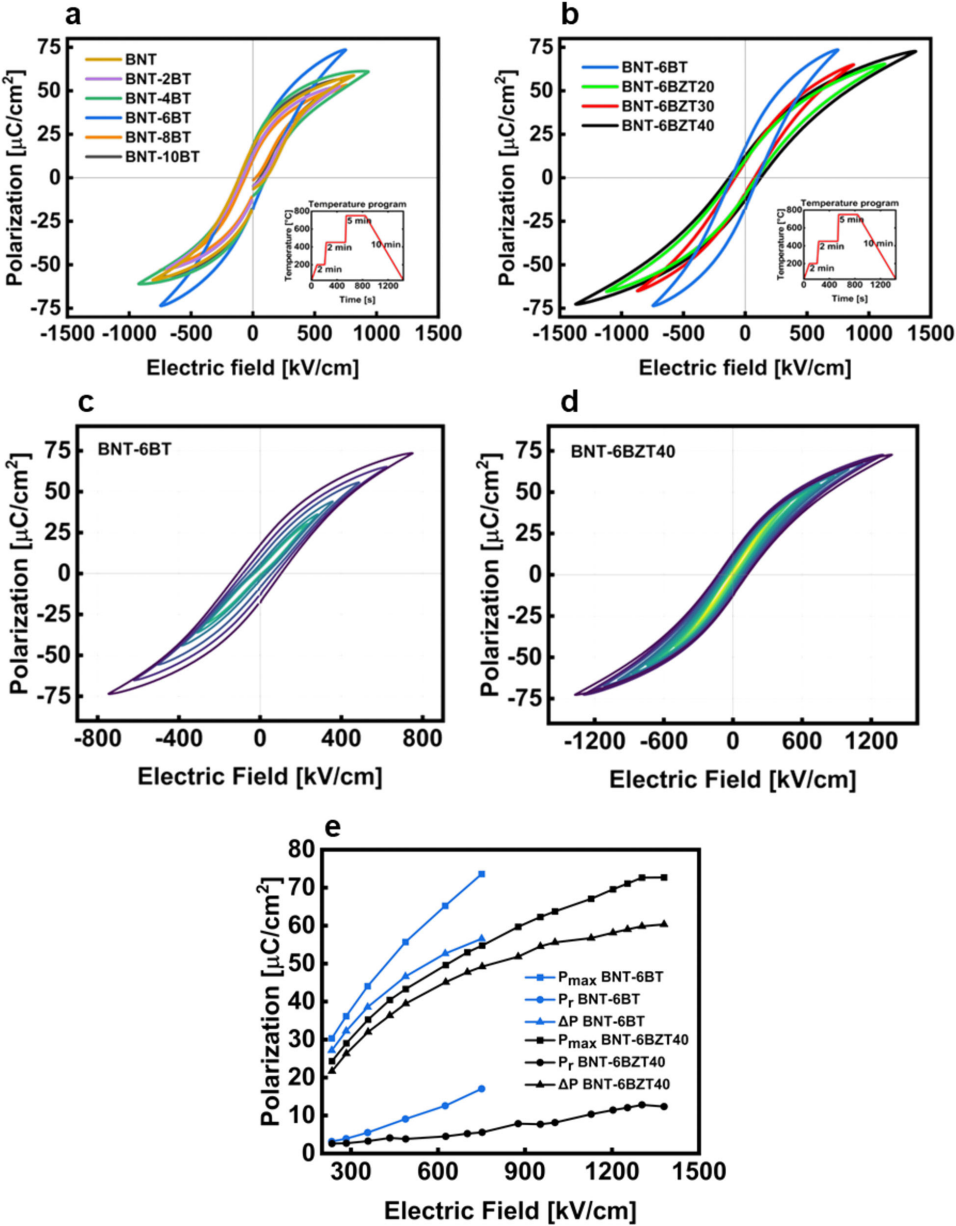
Bipolar Polarization-Electric field hysteresis loops. **a** PE-hysteresis loops at maximum field of BNT-100*x*BT (with *x* = 0, 0.02, 0.04, 0.06, 0.08 and 0.10) thin film series, **b** PE-hysteresis loops at maximum field of BNT-6BZT100*y* (with *y* = 0, 0.2, 0.3, 0.4) thin film series, both with temperature program inlay; nested PE-hysteresis loops of **c** BNT-6BT thin film capacitor and **d** BNT-6BZT40 thin film capacitor and **e** evolution of *P*_*max*_, *P*_*r*_ and ∆*P* of BNT-6BT and BNT-6BZT40.

To characterize the electrical properties of the produced thin films a MIM structure was produced as shown in Fig. 2h. Figure 6 shows the bipolar electric field vs. polarization hysteresis loops (PE-loops) of the produced thin film compositions. All measurements were conducted at room temperature and at a frequency of 1 kHz. In Fig. 6(a) the PE-loops of BNT-100*x*BT are displayed up to the maximum applicable electric field. BNT-6BT clearly shows the best performance, reaching a high maximum polarization of 73 μC cm^−2^ at an electric field of around 750 kV cm^−1^. This increase of 25% in polarization, compared to pure BNT, and 20% compared to adjacent BNT-4BT as well as 37% compared to the adjacent BNT-8BT, respectively, can be explained with the proximity of BNT-6BT to the morphotropic phase boundary^24–27^. The maximization of the maximum polarization is a key aspect to improve the overall energy storage performance of the final device. Therefore, the BNT-6BT composition was chosen for further optimization and chemical modification with Zr, with the aim to further increase the energy storage properties. In fact, another key aspect for improved energy storage is the decrease of the hysteretic losses originating, in ferroelectric compositions like BNT-6BT, from energy dissipation by domain switching. The reduction of hysteretic losses is linked to the disruption of long-range ferroelectric order, resulting in the fragmentation of mesoscopic ferroelectric domains into smaller nanodomains. Zr substitution at Ti site allows achieving this effect, due to the larger size of the Zr cation (0.72 Å)—compared to Ti (0.605 Å)^13^—which prevents B-site off-centering to occur at Zr sites. By introducing a sufficient Zr amount on the B-site (generally, above 35%), ferroelectric long-range order is disrupted, which leads to slimmer hysteresis loops with lower remnant polarization as well as a reduced coercive field. In Fig. 6b the PE-hysteresis loops of BNT-6BZT100*y* are plotted also at the maximum applicable electric field. Here it is visible that the PE-hysteresis loops of the Zr substituted compositions are much slimmer than those of BNT-100*x*BT. The remnant polarization as well as the coercive field are much lower compared to the BNT-6BT thin film, because of the Zr-induced disruption of the ferroelectric long-range order^5,11^. Especially the BNT-6BZT40 composition sticks out as it displays the highest applicable electric field of 1.4 MV cm^−1^ with a maximum polarization of 73 μC cm^−2^ at the maximum field. All Zr substituted films show lower polarization when compared to BNT-6BT at the same field, except BNT-6BZT40 (maximum polarization comparable to BNT-6BT), which can be ascribed to a superior dielectric breakdown strength (87% increase compared to BNT-6BT). In Fig. 6c, d the nested PE-hysteresis loops of the two best compositions of each series are shown, which again confirms that Zr-substitution leads to a slimmer (relaxor-like) PE-loop with higher breakdown strength^11^.

The values of *P*_*max*_ (maximum polarization), *P*_*r*_ (remnant polarization) and ∆*P* (*P*_*max*_ - *P*_*r*_) are plotted over the externally applied electric field in Fig. 6e. From this diagram it is evident that substituting Zr in BNT-6BT not only improved the ∆*P*, but also the dielectric breakdown strength, both of which are relevant to increase the recoverable energy storage density. Despite a lower *P*_*max*_ in BNT-6BZT40, ∆*P* is in the same range of BNT-6BT; this is due to the concurrently low *P*_*r*_ in BNT-6BZT40. These measured values are in good agreement with the assumption that the substitution of Ti with Zr leads to the breaking of the long-range ferroelectric ordering at the perovskite B-site, thus reducing the losses associated with domain switching^5,11,28^. It is also noteworthy that the thin films containing Zr are withstanding higher fields than the films produced without Zr. This can be explained by the better structural properties with a denser and more homogenous microstructure and a flatter top surface (i.e., less inhomogeneities at the interface with the top electrodes), as can be seen in the microstructural characterization (cf. Figs. 2 and 3).

**Fig. 7:**
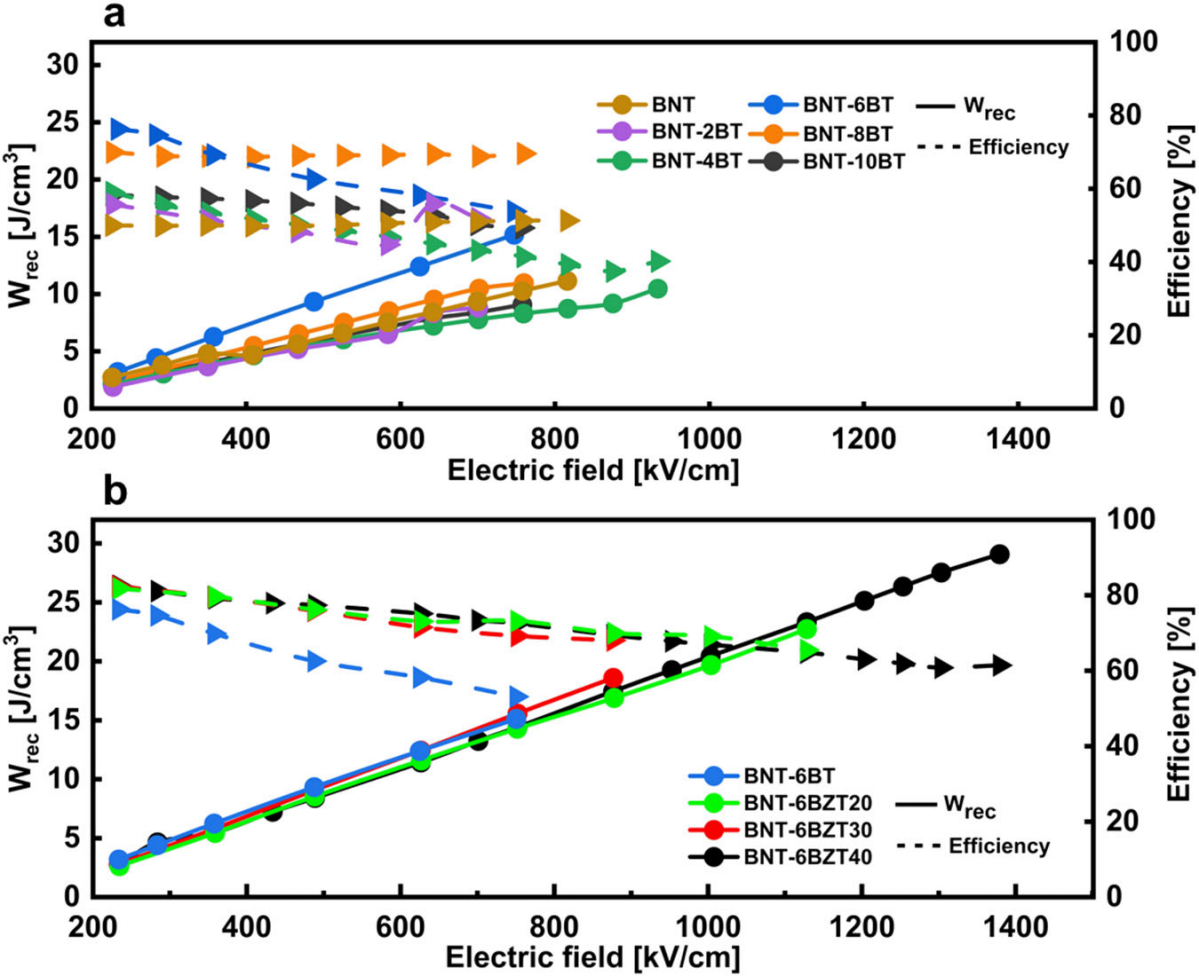
Evolution of the recoverable energy density (*W*_*rec*_) and the efficiency over the electric field. **a** BNT-100*x*BT (with *x* = 0, 0.02, 0.04, 0.06, 0.08 and 0.10) thin film series and **b** BNT-6BZT100*y* (with *y* = 0, 0.2, 0.3, 0.4) thin film series measured at a frequency of 1 kHz and room temperature.

Figure 7a compares the recoverable energy density (*W*_*rec*_) and the efficiency of the BNT-100*x*BT thin film series over the externally applied electric field. As already seen before, BNT-6BT clearly stands out over the whole measurement range, reaching a *W*_*rec*_ = 15 J cm^−3^ and an efficiency of 55%. Figure 7b shows the same graph for the BNT-6BZT100*y* series. At the same electric field, the Zr substituted films show no major difference in energy storage properties, but a slightly improved efficiency, which is ascribed to the lower *P*_*r*_. Interestingly, the maximum applicable electric field before breakdown increases strongly with Zr substitution, reaching 1.4 MV cm^−1^ with *W*_*rec*_ = 29 J cm^−3^ and an efficiency of 60% in BNT-6BZT40, leading to an increase of 93% in recoverable energy storage density compared to the BNT-6BT counterpart.

**Fig. 8:**
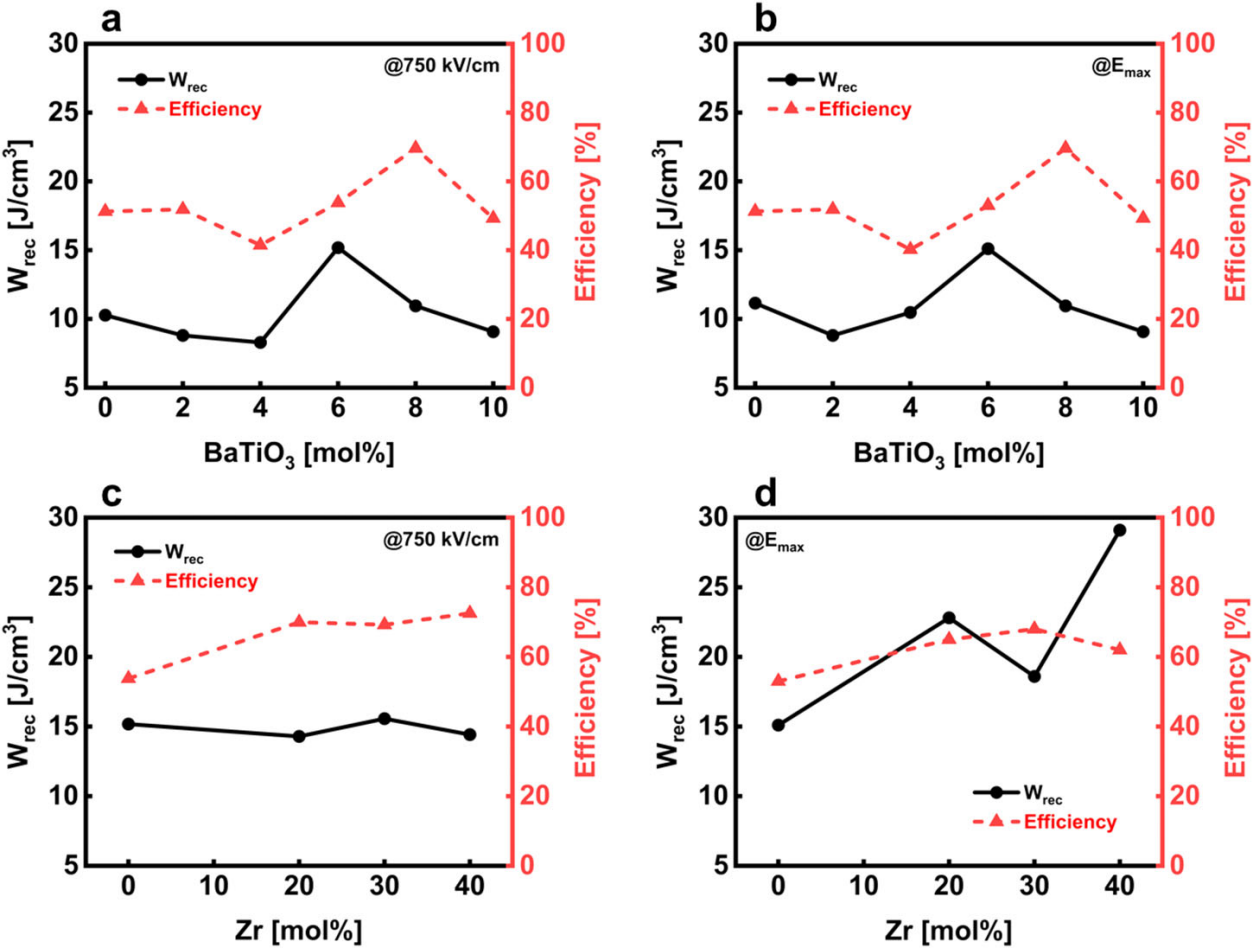
Evolution of the recoverable energy density (*W*_*rec*_) and the efficiency over different amounts of substitution. **a** BaTiO_3_ at a field of 750 kV cm^−1^, **b** BaTiO_3_ at the maximum reachable field, **c** Zr at a field of 750 kV cm^−1^ and **d** Zr at the maximum reachable field.

Figure 8 shows the recoverable energy densities and the efficiencies plotted over the different amounts of BaTiO_3_ and Zr substitution at 750 kV cm^−1^ in (a) and (c), respectively, and at the maximum applied field in (b) and (d) for BNT-100*x*BT and BNT-6BZT100*y*, respectively. The maximum energy storage properties and especially the recoverable energy density are both seen at the morphotropic phase boundary (BNT-100*x*BT with *x* = 0.06) and are underlined by a largely increased polarization (cf. Fig. 8a, b). In BNT-6BZT100*y*, the recoverable energy density appears to stay nearly constant because of the both reduced remnant and maximum polarization from increasing Zr substitution (cf. Fig. 8c). In this system, the large increase in recoverable energy storage density can be ascribed to the optimized microstructure leading to a largely induced breakdown strength, as seen in Fig. 8d, which reports the energy storage properties at the maximum applicable electric field.

**Fig. 9:**
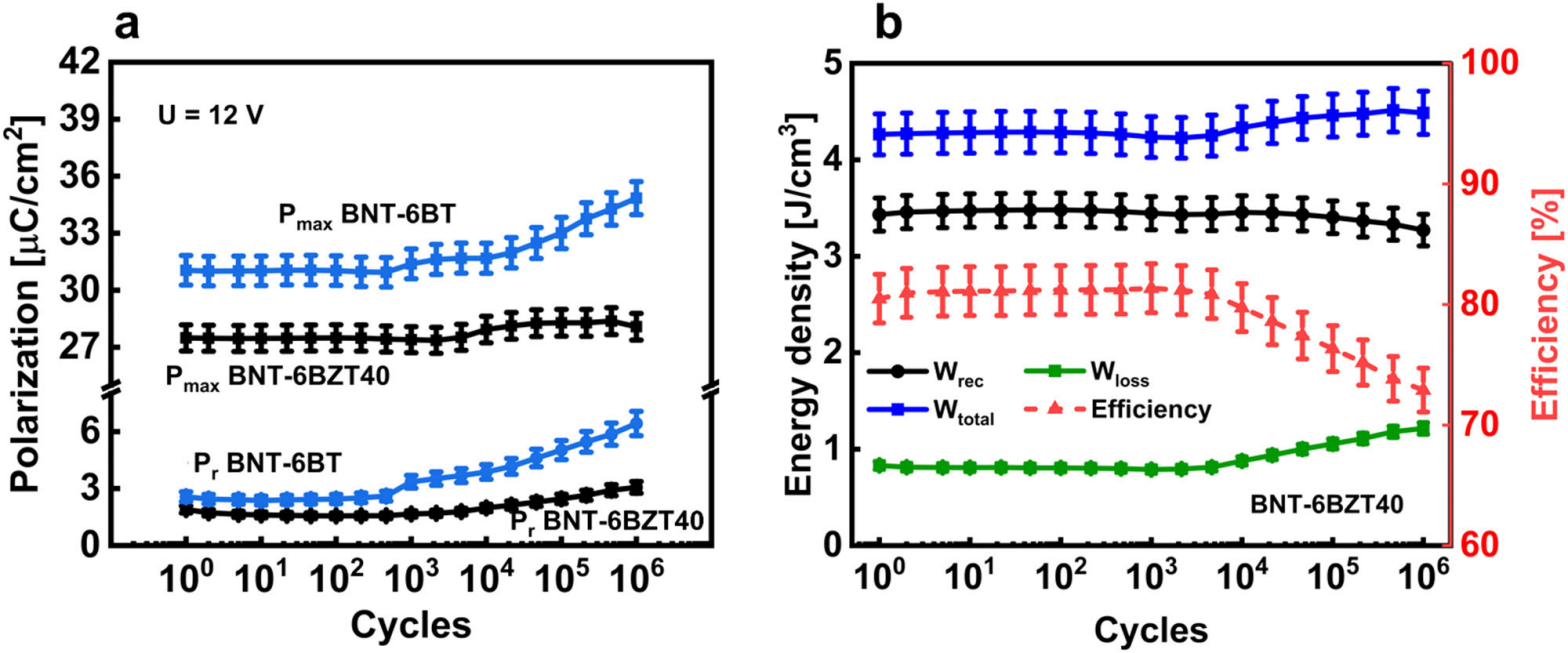
Cyclic stability. **a** Polarization values of cyclic treated BNT-6BT and BNT-6BZT40 thin films, **b** energy storage values of cyclic treated BNT-6BZT40 thin film with estimated error bars.

As mentioned earlier, in our work the top electrodes were deposited with a diameter of 1 mm. This is much larger than the diameter of top electrodes reported on the vast majority of previous works in literature, which use top electrodes with diameters in the range of 200 µm or lower. The rationale behind using smaller top electrodes is to reduce the probability of finding critical defects in the film below the electrodes, which would lead to a significantly lower breakdown strength. Critical defects may originate from impurities, inhomogeneous microstructure or porosity, which are generally exacerbated by the CSD methodology. Larger electrodes, however, are desirable in order to increase the amount of energy that can be stored in the MIM thin film capacitor. In our case, using larger electrode diameters was enabled by the very high quality of the CSD films we produced.

### Electrical fatigue and reliability

The charge-discharge reliability was investigated in BNT-6BT and BNT-6BZT40 (the latter being the most promising composition for application) by applying 10^6^ bipolar cycles to MIM capacitor structures with a frequency of 1 kHz and a voltage of 12 V, representing a typical application scenario for these thin film capacitors. Each decade, three PE-loops were recorded with the same conditions as the electric cycling, to keep track of the variation of the electrical properties upon cycling. The polarization values *P*_*r*_ and *P*_*max*_ and the effective change of the energy storage properties of BNT-6BZT40 and BNT-6BT are shown in Fig. 9. BNT-6BT is stable just up to 10^3^ cycles before the polarization hysteresis starts to inflate, whereas BNT-6BZT40 shows a stable behavior of *P*_*max*_ and *P*_*r*_ up to 10^4^ cycles. Above this number of cycles, a maximum change of +2% is measured in *P*_*max*_ and +50% in *P*_*r*_. Concerning energy storage properties, BNT-6BZT40 showed constant recoverable energy storage density of around 3.4 J cm^–3^ up to 10^4^ cycles, and a maximum relative decrease of 3% in recoverable energy density up to 10^6^ cycles. BNT-based compositions are known to have unstable properties over electric cycles^29^. This is mainly ascribed to oxygen vacancies, which can easily occur in this system because of the volatilization effect of Bi and Na during the heat treatment in processing^5^. This volatilization is partly counteracted by adding excess Bi and Na in the solution before processing. Also the partial transformation from Ti^4+^ to Ti^3+^ during processing can lead to oxygen vacancies’ generation^5^. The aforementioned early degradation observed in BNT-6BT can thus be ascribed to a combination of these mechanisms leading to oxygen vacancy generation. These effects are counteracted also by substituting on the B-site with 1 mol% Mn, as explained above^30^. As seen from the better long-term fatigue behavior of BNT-6BZT40, Zr substitution seems to produce a beneficial effect on the stability of electrical properties. Zr substitution, in fact, has been shown to have a positive effect on the thin film microstructure, such as higher density with less porosity and an improved dielectric-metal interface^5^.

### Virtual device model

**Fig. 10:**
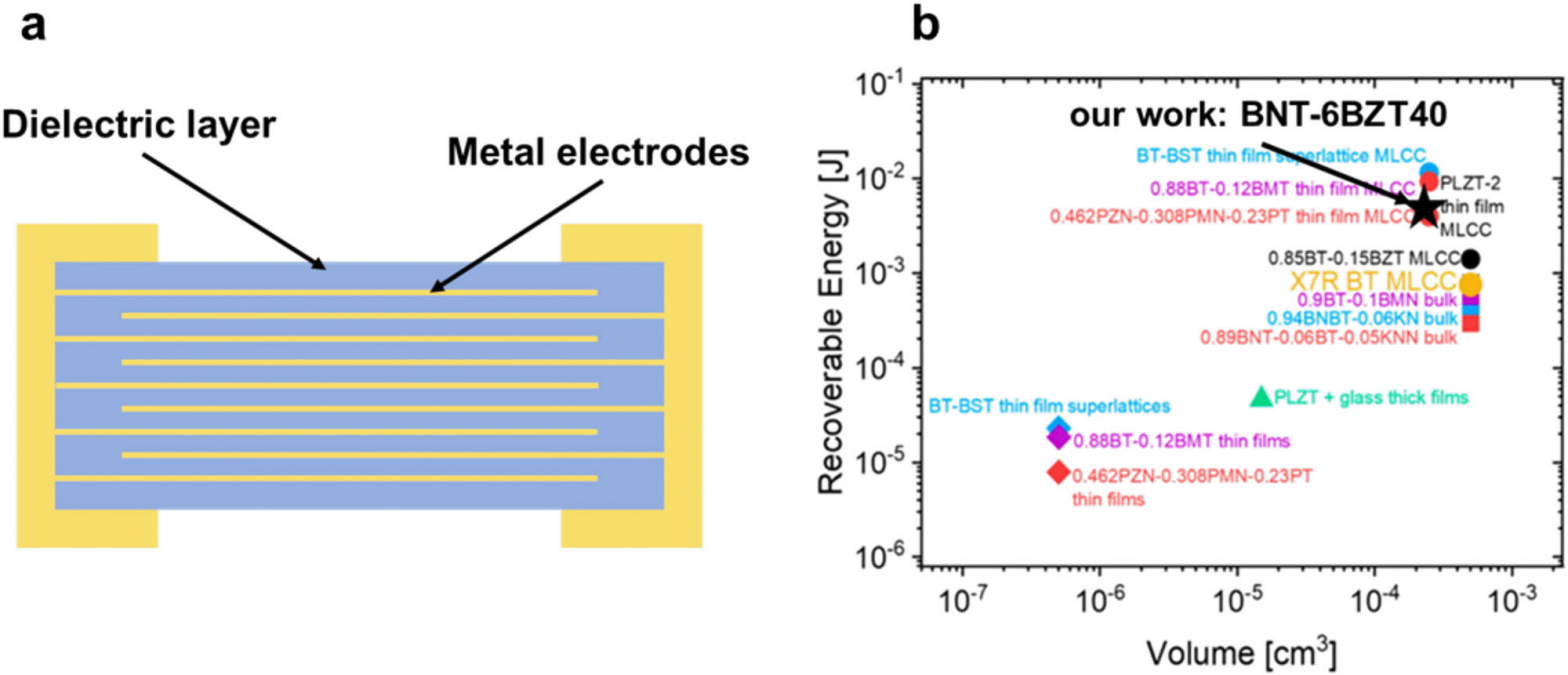
Virtual device model. **a** Scheme of the virtual device model of a BNT-6BZT40 thin film capacitor structure. **b** Recoverable energy density at the maximum applied electric field for some selected relaxor compositions in bulk, thick-film multilayers and thin-film multilayers. Values for MTFCs were extrapolated from the respective *J*_*r*_ values of single-layer thin films assuming an MLCC consisting of 500 layers with 1 µm thickness and 0.5 × 1 mm^2^ area (**b** modified from ref. ^2^); the best composition BNT-6BZT40 of this work is marked with a star.

To fully use the advantages of thin films for high recoverable energy density and efficiency while enabling substantial energy storage in a capacitor, the implementation of a multilayer architecture is essential. A MTFC with the same volume of a typical multilayer ceramic capacitor (MLCC) would thus be able to store at least two orders of magnitude higher energy than the MLCC in virtue of the higher recoverable energy density^2^. Such capacitor would ideally serve as sole energy storage element in energy autonomous IoT devices. In order to illustrate the benefits of such MTFC, we constructed here a virtual device model by calculating the energy stored in an MTFC using BNT-6BZT40 as basis material because of its both superior energy storage and microstructural characteristics. The virtual device, schematically shown in Fig. 10a, consists of a multilayer of 500 thin film layers, each with 1 µm thickness (comparable to thin MLCC layers) and an effective area of 0.5 mm^2^. Such MTFC would have a total energy-storing active volume of 0.25 mm^3^ and would be able to deliver 7.25 mJ recoverable energy. In IoT sensors, sensing events, transmission events, and sleep modes have energy requirements in the mJ range, making such MTFC stacks suitable as energy supply devices for IoT energy autonomous sensors. Figure 10b gives an overview of virtual MTFC devices based on literature values of the recoverable energy density for selected relaxor compositions and the performance of the composition presented in this work^2^. This clearly shows that the thin film produced in our work has superior performance to many other compositions, and thus can be considered as a promising candidate for capacitive energy storage for IoT applications.

## Conclusion

In this work we successfully fabricated BNT-100*x*BT (with *x* = 0, 0.02, 0.04, 0.06, 0.08 and 0.10) and BNT-6BZT100*y* (with *y* = 0, 0.2, 0.3, 0.4) thin films with a thickness of 200 nm on platinized Si-wafers from CSD for high energy storage MIM-structured capacitors. The films exhibit a dense and uniform microstructure. Notably, the increase in surface roughness caused by BaTiO_3_ substitution was effectively mitigated by the introduction of Zr, which significantly enhanced the dielectric breakdown strength. The maximum energy storage properties of the two thin film series were 15 J cm^−3^ and an efficiency of 55% for BNT-6BT and 29 J cm^−3^ and an efficiency over 60% for BNT-6BZT40. The Zr substituted compositions showed slimmer PE-loops due to the disruption of long-range ferroelectricity. The Zr substituted composition BNT-6BZT40 also shows enhanced charge-discharge reliability, making it a promising candidate for energy storage capacitors. This is supported by a virtual device model for application of this composition as MTFC in an IoT-sensor. It was shown that a BNT-6BZT40 MTFC would be able to deliver a total of 7.25 mJ, making it suitable for powering events of an IoT-sensor.

## Methods

### Sample characterization

**Fig. 11:**
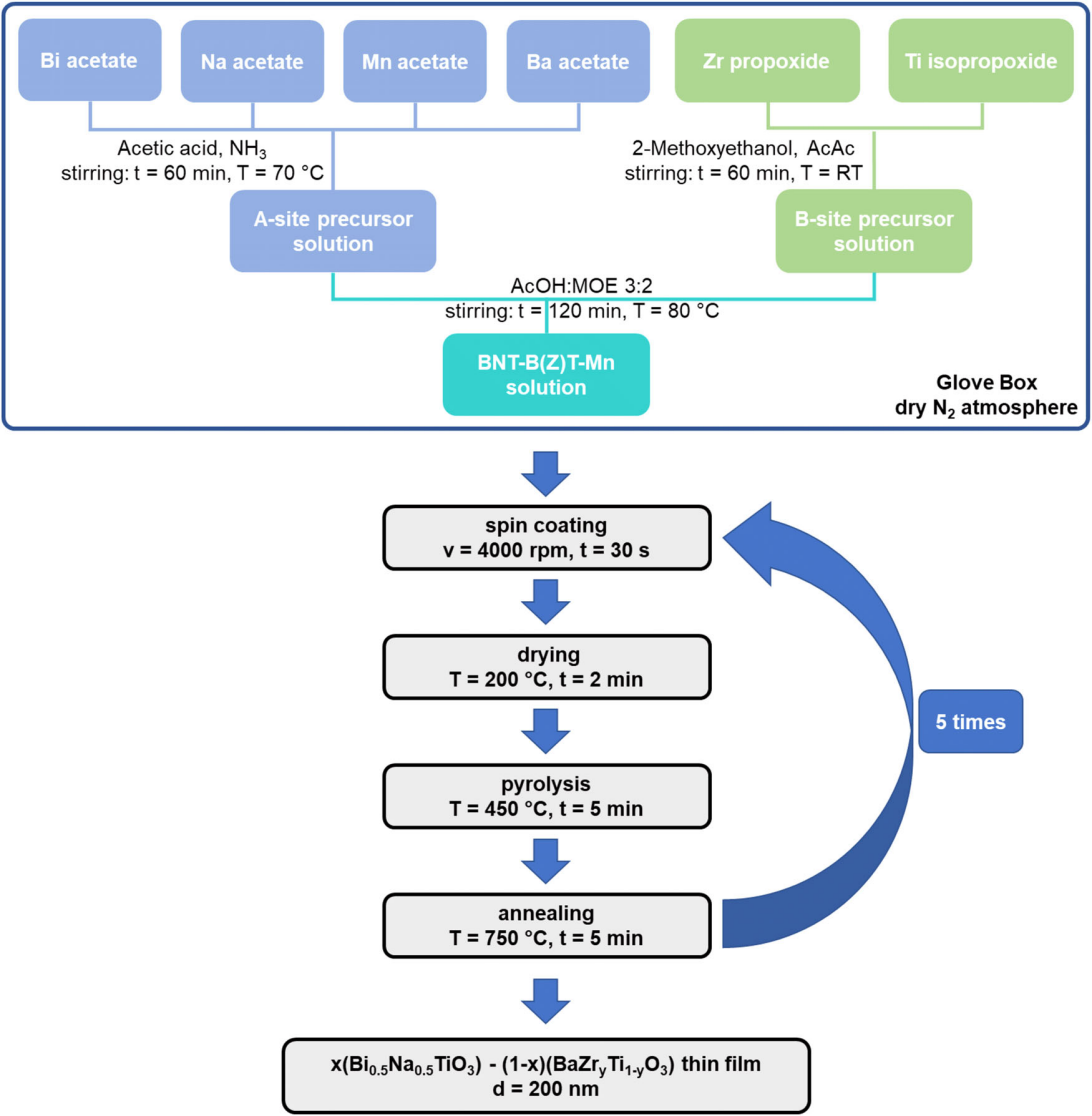
Experimental procedure for the synthesis and fabrication of BNT-100*x*BT and BNT-6BZT100*y* thin films via chemical solution deposition.

The investigated thin films were prepared by CSD with organic solvents. Figure 11 schematically illustrates the performed experimental procedure for the synthesis of the solutions as well as the deposition of the thin films of stoichiometric composition (*1-x*)Bi_0.5_Na_0.5_TiO_3_–*x*BaTiO_3_ (with *x* = 0, 0.02, 0.04, 0.06, 0.08 and 0.10; BNT-100*x*BT) and 0.94Bi_0.5_Na_0.5_TiO_3_–0.06BaZr_*y*_Ti_*1-y*_O_3_ (with *y* = 0, 0.2, 0.3, 0.4; BNT-6BZT100*y*) with 1 mol% Mn substituted on the B-site^30^. To avoid potential contamination of the environment, and to protect hygroscopic precursors from ambient humidity, the complete solution synthesis route was performed under a dry N_2_ atmosphere in a glove box (Sylatech, Germany). As starting materials bismuth(III) acetate (Alfa Aesar, purity 99%, Bi(CH_3_COO)_3_), sodium acetate (Carl Roth, purity 99%, Na(CH_3_COO)), barium(ll) acetate (Carl Roth, purity 99%, Ba(CH_3_COO)_2_), titanium(IV) isopropoxide (Sigma Aldrich, purity 97%, C_12_H_28_O_4_Ti), zirconium(IV) propoxide solution (Sigma Aldrich, purity 70% in 1-propanol, Zr(OCH_2_CH_2_CH_3_)_4_) and manganese(II) acetate tetrahydrate (Sigma Aldrich, purity 99%, Mn(CH_3_CO_2_)_2_*4H_2_O) were selected. Acetic acid (Carl Roth, purity 100%, C_2_H_4_O_2_), 2-methoxyethanol (Carl Roth, purity 99%, C_3_H_8_O_2_) were used as solvents; acetylacetone (Sigma Aldrich, purity 99%, C_5_H_8_O_2_) and ammonia solution (Merck, purity (28-30)%, NH_4_OH) were used as complexing agent and for pH adjustment, respectively.

Both solutions followed a similar synthesis route, however, BNT-6BZT100*y* needed some minor adjustments for the preparation of the B-site solution to incorporate zirconium propoxide precursor. As explained in the next section, BNT-6BT was chosen as starting composition for BNT-6BZT100*y* because this composition offers the highest energy storage properties within the binary BNT-BT system due to the presence of the morphotropic phase boundary^11^. To compensate the volatility of some precursors, an appropriate excess of the used metal cations was used in the solutions. For the BNT-100*x*BT and the BNT-6BZT100*y* A-site solutions, bismuth acetate (2% excess), barium acetate (5% excess), sodium acetate (10% excess) and manganese acetate got dissolved in acetic acid with the additional help of ammonium and stirred for 60 min at 70 °C. The B-site solution of BNT-100*x*BT was prepared by complexing titanium isopropoxide with acetylacetone in a molar ratio of AcAc:Ti = 2:1 and then dissolving it in 2-methoxyethanol after 5 min of stirring at room temperature. The B-site precursor solution of BNT-6BZT100*y* was prepared by adding zirconium propoxide after another 5 min of stirring. Afterwards, the B-site solutions were stirred for 60 min at room temperature. Then, the Ba-Na-Bi-Mn solution was added drop by drop under constant stirring to the Ti solution and the Ti-Zr solution for BNT-100*x*BT and BNT-6BZT100*y*, respectively. After another 2 h of mixing at 80 °C the final BNT-100*x*BT and BNT-6BZT100*y* solutions were prepared by adding acetic acid and 2-methoxyethanol in a molar ratio of AcOH:2-MOE = 3:2 to obtain 0.3 M final precursor solutions ready for deposition.

Before starting the deposition procedure, the used substrates were first rinsed with deionized water, ethanol and isopropanol, after which they were spin-cleaned and dried on a hot plate for 5 min at 150 °C. The synthesized solutions were deposited onto (1 × 1) cm^2^ platinized silicon substrates (Pt/TiO_2_/SiO_2_/Si, SINTEF, Norway) with classical spin coating (SPIN150i, APT GmbH, Germany) in a laminar flow box (BioWizard, KOJAIR, Finland). To realize uniformly distributed, homogenous films the deposition was performed for each layer for 30 s with a constant velocity of 4000 rpm.

Thereafter, a rapid thermal annealer (MILA-5050, Advance Riko, Japan) was used for crystallization of the thin films. The heat treatment was performed in an oxygen atmosphere, comprising a drying step at 200 °C for 2 min (∆*T* = 2 °Cs^-1^), an organic burn-off at 450 °C for 5 min (∆*T* = 10 °C s^−1^) and an annealing step at 750 °C for 5 min (∆*T* = 25 °C s^−1^). To obtain a total thin film thickness of 200 nm the above-described steps of deposition and thermal treatment were repeated 5 times.

To realize a capacitor-like structure and evaluate the energy storage properties of the two fabricated material systems BNT-100*x*BT and BNT-6BZT100*y*, gold top electrodes with a thickness of 200 nm and a diameter of 1 mm were deposited with an e-beam assisted evaporation device (Hex Series TAU, Korvus Technology, United Kingdom). Prior to deposition, the samples were spin-cleaned and dried in the same way as the substrates before.

### Characterization

The electrical properties, including polarization vs. the electric field (PE) loops and fatigue measurements, were recorded with an aixACCT TF3000 analyzer (Germany) at room temperature in air.

The microstructure of the thin films was investigated with a Scanning Electron Microscope (SEM, Zeiss AURIGA®-CrossBeam® dual-beam, Zeiss, Germany). The images were analyzed using the software Fiji^31^. At least 100 grains per sample were used for evaluation of the grain size distribution. Atomic Force Microscopy characterization was performed using CoreAFM (AFM, Nanosurf, Liestal, Switzerland) under ambient conditions. Non-contact (tapping) mode was used for image acquisition. The scan parameters were initialized with a value of 5% of the nominal force and an acquisition time of 0.78 s on an area of (10 × 10) μm. Images were processed using the Gwyddion program^32^. For further structural analysis, a Raman spectrometer (WITec alpha300R, WITec GmbH, Germany) was used, with an excitation source of 532 nm wavelength solid-state laser with 10 mW power, 1800 gr mm^−1^ grating and a 100x EC Epiplan-Neofluar DIC objective (ZEISS, Germany). For the analysis of the phases in the thin films, especially to detect phase purity and crystallographic information, grazing incidence X-ray diffractometry (GI-XRD, D8-Discover Series II, Bruker, Germany) was performed using Cu-Kα radiation.

## Data Availability

All data are available from the corresponding authors upon reasonable request.
